# Laser Speckle Imaging: A Novel Method for Detecting Dental Erosion

**DOI:** 10.1371/journal.pone.0118429

**Published:** 2015-02-13

**Authors:** Nelson H. Koshoji, Sandra K. Bussadori, Carolina C. Bortoletto, Renato A. Prates, Marcelo T. Oliveira, Alessandro M. Deana

**Affiliations:** 1 Dep. of Biophotonics, Nove de Julho University (UNINOVE), São Paulo, Brazil; 2 School of Dentistry, Nove de Julho University (UNINOVE), São Paulo, Brazil; 3 School of Information Technology, Nove de Julho University (UNINOVE), São Paulo, Brazil; 4 School of Engineering, Nove de Julho University (UNINOVE), São Paulo, Brazil; University of New Mexico HSC, UNITED STATES

## Abstract

Erosion is a highly prevalent condition known as a non-carious lesion that causes progressive tooth wear due to chemical processes that do not involve the action of bacteria. Speckle images proved sensitive to even minimal mineral loss from the enamel. The aim of the present study was to investigate the use of laser speckle imaging analysis in the spatial domain to quantify shifts in the microstructure of the tooth surface in an erosion model. 32 fragments of the vestibular surface of bovine incisors were divided in for groups (10 min, 20 min. 30 min and 40 min of acid etching) immersed in a cola-based beverage (pH approximately 2.5) twice a day during 7 days to create an artificial erosion. By analyzing the laser speckle contrast map (LASCA) in the eroded region compared to the sound it was observed that the LASCA map shifts, proportionally to the acid each duration, by: 18%; 23%; 39% and 44% for the 10 min; 20 min; 30 min and 40 min groups, respectively. To the best of our knowledge, this is the first study to demonstrate the correlation between speckle patterns and erosion progression.

## Introduction

Laser speckle imaging is a diagnostic technique in which the features of scattered coherent light are explored. At first considered noise, the image of the scatter pattern actually contains information on the microstructure and micro-movements of the surface of a given tissue. By employing statistical analysis of the temporal and spatial fluctuations in the light scattered by microstructure dynamics and heterogeneities, it is possible to extract information on the dynamics of the abdominal wall in rats, pulp vitality in teeth and cerebral blood flow.

The ability of laser speckle imaging to allow the evaluation of dynamic features in tissues using a non-invasive, non-destructive cost-effective, real-time method has stimulated the academic community to focus efforts on the study of this method in the time domain (dynamic speckle analysis). However, the analysis of speckle patterns in the spatial domain also contains information on the microstructure and heterogeneities of the surface, which can be explored by applying the proper statistical analysis. Deana *et al*. [1] describes a method to enhance the contrast between sound and decayed tooth tissue through the study of laser speckle pattern shifts in the spatial domain.

Tooth erosion is defined as change in the ultrastructure of the enamel, which is currently assessed only using clinical diagnostics. Erosion is highly prevalent, affecting more than 50% of the population under five years of age and up to 77% of elderly individuals (> 60 years of age) [2, 3]. Tooth wear is a natural process caused by friction during chewing and brushing as well as exposure to acidic foods and beverages. This process becomes pathological when the degree of destruction compromises the function and esthetics of the teeth, with the emergence of sensitivity, which can range from mild discomfort to the impossibility of ingesting certain substances [4–6].

Tooth erosion in all age groups has gained importance in recent years due to the pace of modern life, which often leads to the replacement of fresh foods by industrialized foods containing acidic ingredients and conservatives. Indeed, there is a close association between the increase in tooth erosion and the ingestion of acidic foods and beverages [7]. Moreover, tooth erosion is associated with certain adverse health conditions, such as acid reflux, bulimia and vomiting due to excessive alcohol intake, as gastric fluid is highly acidic [8,9].

Demineralization of the teeth is generally caused by an acidic substance with a lower pH than the critical threshold for the enamel (5.5) and dentin (4.5), which can dissolve hydroxyapatite crystals [9, 10]. The main signs and symptoms of demineralization are pain, discoloration, transparency, cracking and the formation of pits, with microscopic changes to the tooth surface.

A large number of studies have investigated the prevention of erosion lesions [2–12], but the loss of dental tissue is irreversible. Thus, early diagnosis is crucial to minimizing the amount of damage and plays an important role in the decision-making process of dentists.

The aim of the present study was to detect and quantify small changes in the microstructure of teeth using laser speckle imaging analysis to assess dental tissue demineralization.

## Materials and Methods

### Ethics

The Brazilian law number 11.797 (that regulates animal procedures) published on October 8^th^, 2008, paragraph 3^rd^, article 3 defines animal experiments as: “procedures made on live animals”, therefore since all samples were obtained *post mortem* from disposable parts of animals grown for commercial slaughter purposes at Frigobet, this work don’t require approval from the animal ethics committee.

### Sample preparation

Using the method proposed by Shellis et al.[13], Schluter et al. [14], Young et al. [15] and Cheng et al.[16], 32 fragments of the vestibular surface of bovine incisors were obtained. Two fragments measuring approximately 6 x 6 mm² were embedded in each sample holder (PVC tube) with acrylic resin with the enamel exposed, horizontal and parallel. Each sample was polished for 60 seconds using wet sandpaper with different degrees of coarseness (400, 600, 1000 and 1200, Buehler, UK). A felt disk with a diamond paste (3M, USA) was then used for polishing. Each fragment was divided into two parts, one of which was protected with nail polish (classified as sound tissue) and the other was left exposed and submitted to chemical corrosion (classified as eroded tissue).

For the erosion challenge, the samples were divided into four groups and immersed in 30 ml of a cola-based beverage (pH approximately 2.5) at room temperature (approximately 25°C). Immersion was performed twice a day over seven consecutive days using the following experimental protocol:

- Group 1 (n = 8) - 10 minutes;
- Group 2 (n = 8) - 20 minutes;
- Group 3 (n = 8) - 30 minutes;
- Group 4 (n = 8) - 40 minutes.

After each challenge, the samples were rinsed with de-ionized water for 20 seconds, dried at room temperature and stored in a humid environment until the subsequent etching acid. Two outliers were excluded from group 2 [13–16].

### Laser speckle imaging

**Fig 1:**
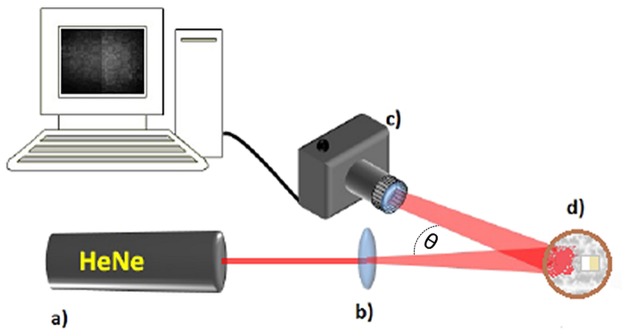
Schematic diagram of laser speckle imaging system. (a) HeNe laser emitting at 633 nm; (b) Beam expander lens; (c) CMOS camera; (d) samples.

Fig. 1 shows the schematic diagram of the laser speckle imaging system. The surfaces of each sample were imaged under a coherent light illumination at normal incidence. A HeNe laser (Uniphase, USA) emitting at 633 nm with 40 mW of continuous wave power was used. The bean was expanded by a f = 100 mm lens (K&F concept, China) achieving a circular spot size with 6 mm in diameter. The samples were than imaged using a CMOS sensor with 23.7 mm X 15.3 mm (4752 x 3168 pixels; pixel pitch = 4.99 μm) (Canon EOS Rebel T1i camera fitted with a macro 100 mm Canon lens, Japan) and stored. The photometric parameters were: exposure time = 1/200 s; f/ 29; ISO 100 and the camera was placed at an angle θ < 10° with the laser (Fig. 1). No data binning was performed by the camera.

**Fig 2:**
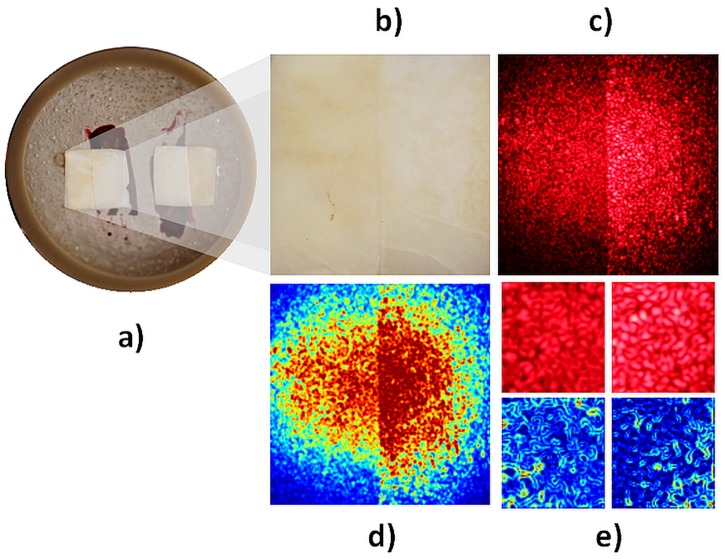
Procedure for acquisition and analysis of speckle images. (a) sample; (b) image under white light; (c) image under coherent light; (d) image with false colors; (e) LASCA method.

The samples were placed inside a plastic tube (Fig. 2a). Each sample was imaged under white (Fig. 2b) and laser (Fig. 2c) illumination. Fig. 2d presents the speckle imaging mean (n = 4x4) in which a false color algorithm was applied to increase the visual contrast. Each raw image was manually trimmed to obtain a 700 x 700 pixel image (Fig. 2e) containing the region of interest with a sample of the sound tissue (right) and a sample of eroded tissue (left).

The methods currently used to extract information from speckle images are mainly based on the analysis of pixel intensity (brightness) of the image [1,17,18]. The images were analyzed by a custom software written (by the authors) in Python Language Reference (version 2.7,) based on the theoretical analysis of speckle images presented in ref. [19]. According to [19], the scattered amplitude fields are randomly distributed and the intensity values follow a negative exponential distribution (1) [19]:

$$P(I)=e^{\left(-\frac{I}{<I>}\right)}$$ (1)

in which <I> is the mean intensity given by (2):

$$<I>=\frac{\sum_{i=1}^{n}I}{n}$$ (2)

and the standard deviation of the pixel intensity is (3):

$$\sigma =\sqrt{\frac{\sum_{i=1}^{n}{\left(I_{i}-<I>\right)}^{2}}{n-1}}$$ (3)

in which *n* is the sample size.

High spatial resolution is desirable when analyzing oral tissues. Therefore, analysis of variance should be performed on small samples, but without compromising the statistical accuracy. Extrapolating data obtained from Deana et al. [1], a sample size of *n* = 4 x 4 was used in the present study, resulting in a 175 x 175 pixel image (Fig. 2d).

Laser speckle contrast analysis (LASCA) is another statistical method that is usually combined with the extraction of movement information from a spatial image and it is calculated as shown (4):

$$C_{i,j}=\frac{\sigma_{i,j}}{<I_{i,j}>}$$ (4)

in which the contrast *C* is a number ranging between 0 and 1. Low contrast values denote fast moving particles, whereas high values denote slow moving particles [20].

Although usually associated with movement, contrast analysis also reveals interesting features in the spatial domain, as will be demonstrated in this paper, whereby the contrast map of the images is also studied.

To avoid heterogeneity in laser beam intensity, the contrast map was calculated only for the central portion of the image, where the illumination intensity is homogeneous (Fig. 2e).

### Statistical analysis

The data were found to present normal distributions (Shapiro-Wilk; p > 0.05), therefore analysis of variance was used in order to compare multiple groups followed by Tukey as post-hoc. The correlation between the acid etching duration and the LASCA ratio was assessed by the Pearson’s test. The inferential analysis was performed by BioEstat 5.3 (Brazil) and the significance level is set at α = 0,05.

## Results and Discussion

**Fig 3:**
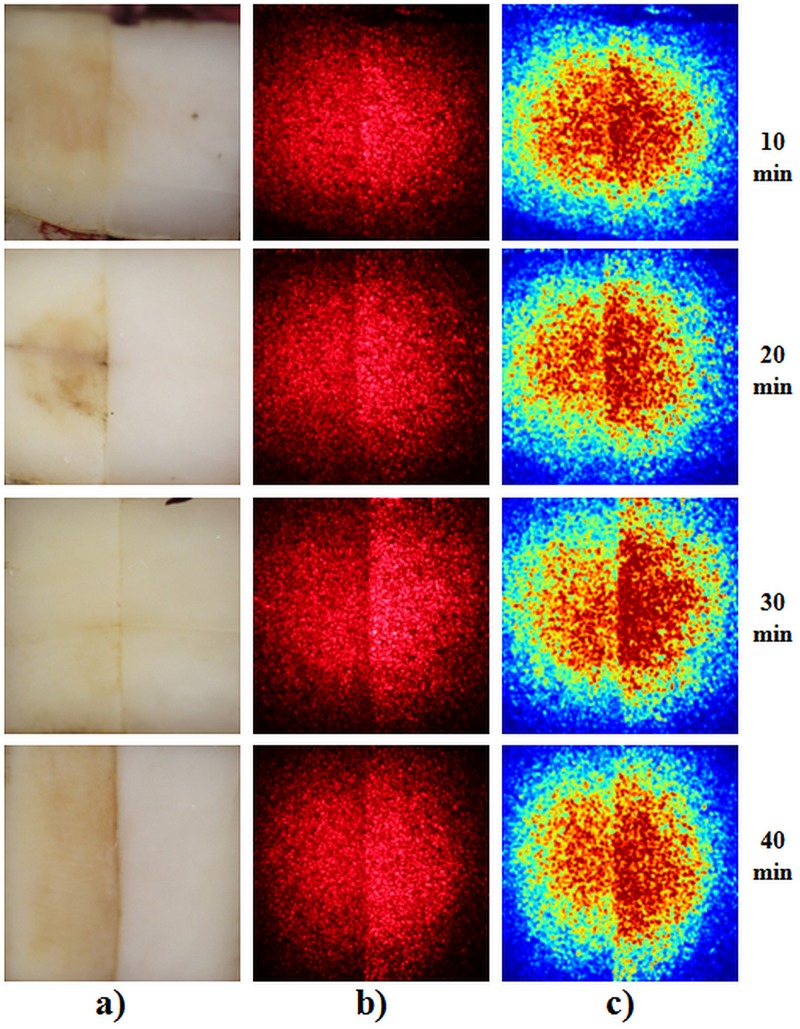
Sample; (a) white light image; (b) coherent light image; (c) false color image with averaged intensity.

The laser speckle images demonstrate that it is possible to acquire information on the microstructure of the enamel and detect minimal changes, such as early non-carious lesions. Fig. 3a shows a representative sample from each group under white illumination. Although there are visible stains in the left portion of each sample due the dye from the cola beverage, structural changes are difficult to assess with the naked eye.

In Fig. 3b, each sample was imaged under laser illumination. The images were then averaged (n = 4 x 4) and a false color algorithm was applied to facilitate the visualization.

**Fig 4:**
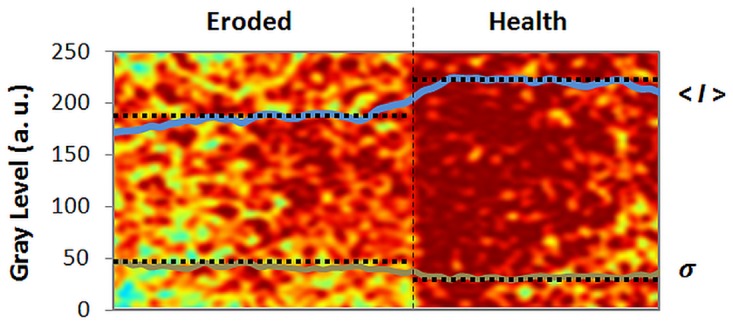
Average intensity and standard deviation of a typical laserspeckle image of tooth enamel.

All samples exhibited lower average intensity of the backscattered light on the eroded tissue, which is seen on the left side of Fig. 3a and b, in comparison to sound tissue (right side). Fig. 3c presents the speckle imaging mean (n = 4x4) in which a false color algorithm was applied to enhance the visual contrast. Moreover, the standard deviation of each sample was larger in the eroded tissue in comparison to the sound tissue (Fig. 4). Both effects are related to the heterogeneity induced in the microstructure of the enamel by the demineralization process, which increases the interprismatic spaces, exposing the top of the prism, which has greater porosity [21]. This increases the number of scattering centers, thereby increasing the light scattering, but mineral loss also reduces the backscattering coefficient. This opaque surface therefore results in a larger standard deviation and less average intensity in laser speckle images (Fig. 4).

**Fig 5:**
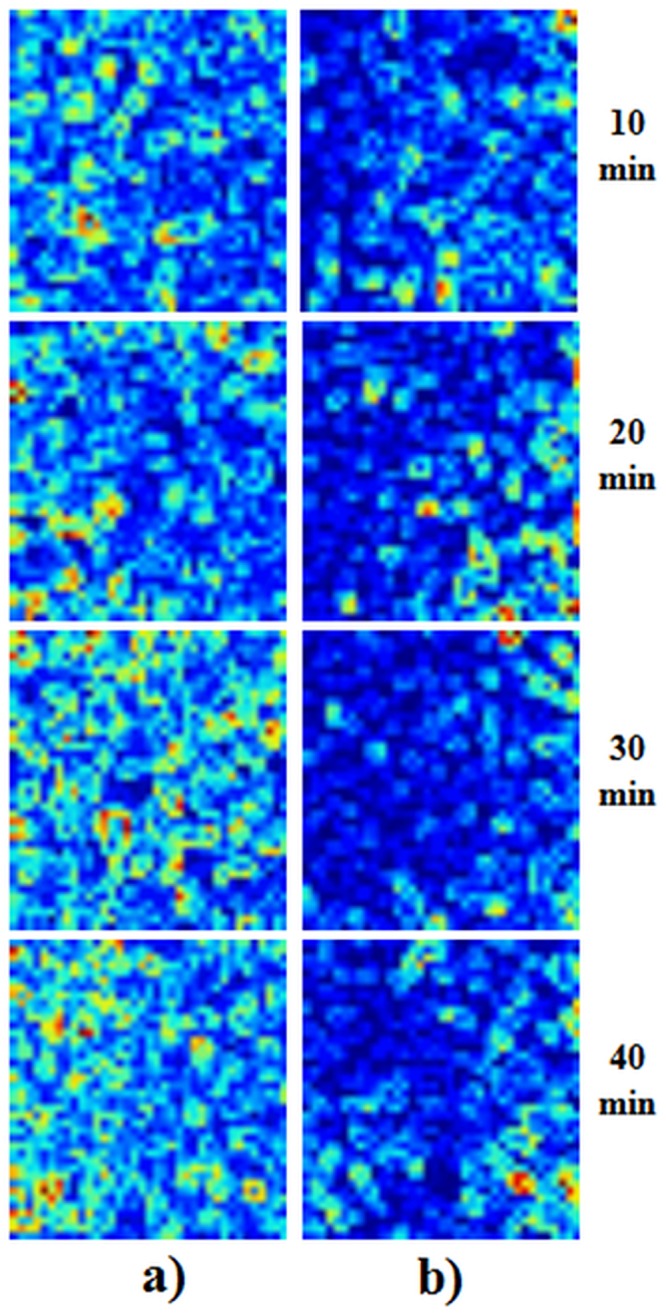
Typical LASCA maps of; (a) eroded tissue; (b) sound tissue.

To differentiate the sound and eroded tissues, contrast analysis was performed of the speckle patterns in the images. Since this analysis is, in its essence, the ratio of the standard deviation and average intensity, the LASCA map of the lesion is generally higher than in sound tissue. This phenomenon is demonstrated in the LASCA maps in Fig. 5, which show the greater prevalence of dark blue on the right side, indicating sound tissue, and lower prevalence on the left side, indicating eroded tissue.

The contrast ratio of the LASCA map of the sound and eroded tissue in each sample is also analyzed according to (5) [22]:

$$1-\frac{<C_{sound}>}{<C_{lesion}>}$$ (5)

**Fig 6:**
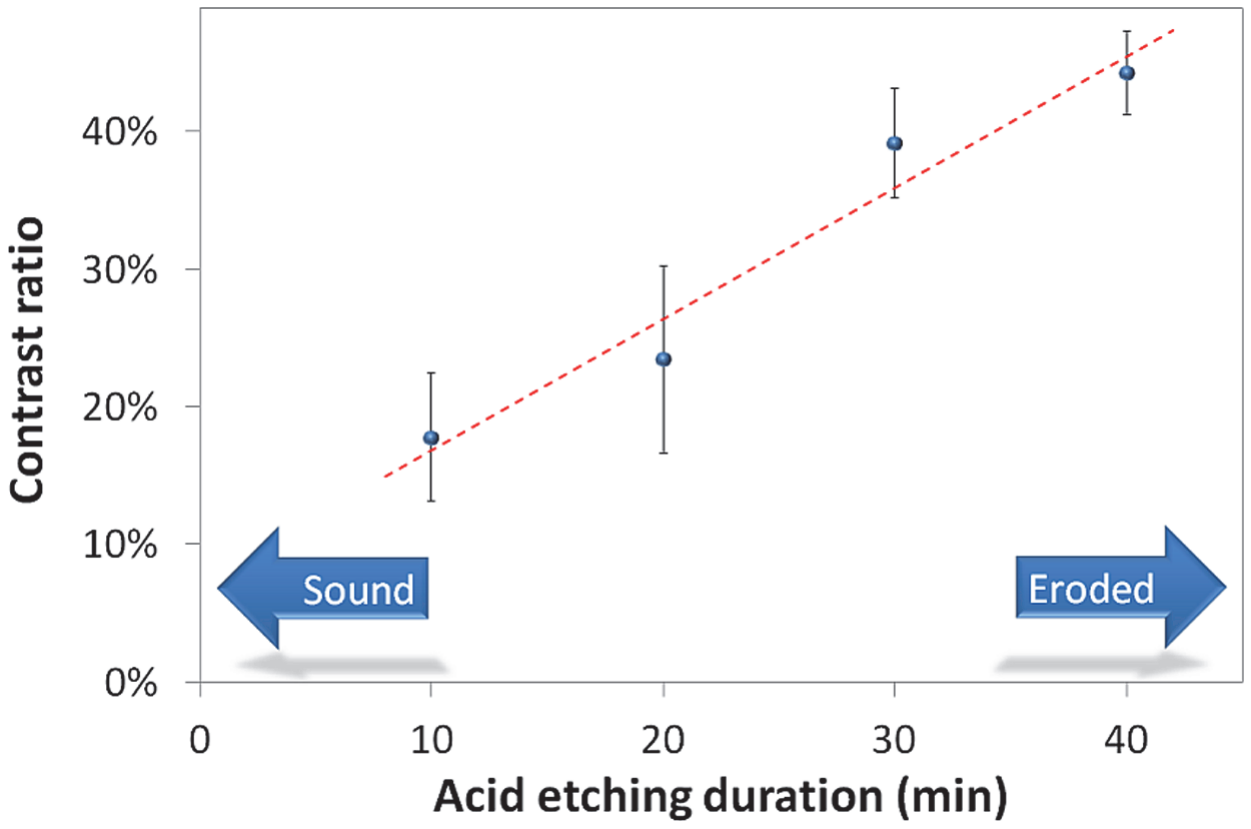
Contrast ratio x acid etching duration (equal letters means statistical difference). The arrow means the group statistically differs from the reference contrast ratio 0%.

Plotting the average contrast ratio for each group against its acid etching duration, a correlation is found between the speckle signal and demineralization process (Fig. 6).

This contrast ratio demonstrates the lower LASCA signal from the eroded tissue lesion in comparison to the sound region. Fig. 6 demonstrates that the contrast ratio (CR) of the speckle images is sensitive to even small changes in the microstructure of the surface.. Although the difference between 10 and 20 minutes; and between 30 and 40 minutes data points are not statistically significant, the overall trend would tend toward a linear correlation with the etching acid duration. (Pearson’s coefficient r = 0.9737, p = 0.0263)

Ten minutes of acid etching in a cola-based beverage resulted in a contrast ratio of 18%, meaning that the LASCA signal originating from the lesion was 18% lower than that from the sound region which, at the significance level α = 0.05, is statistically different from the 0% reference value (p < 0.0001). For the groups 20, 30 and 40 min of etching acid, the contrast ration also statistically differs from the reference value (p < 0.0001). These data indicates the contrast ratio is strongly correlated to the etching acid, correctly detecting the demineralization due to the erosion process even for process as initial as 10 min of acid etch.

At 30 min of acid etching, the contrast ratio increased to 39%, which statistically differs from 10 min (p = 0.0195) and from 20 min (p = 0.0149. The 40 min group, does not statistically differs from the 30 min group but, it is different in comparison with the 10 min group and 20 min group (p = 0.0095 and p = 0.0077, respectively). This demonstrates the proposed method is capable of more than just detect—it also quantifies—the erosion progress thus this process provides an objective way of analyzing the disease progression.

## Conclusions

Erosion is highly prevalent in people of all ages. However, an objective diagnostic procedure is still needed, thus the study of the laser speckle imaging for tooth enamel may provide the first low cost objective diagnostic method for this disease.

The analysis of laser speckle imaging in the spatial domain is a powerful diagnostic technique that provides information on the surface microstructure. To the best of our knowledge, this is the first study to demonstrate it is possible to analyze information on the microstructure of tooth enamel after an acid etching procedure using patterns and LASCA maps. In an erosion model, these patterns are associated with mineral loss from the enamel.

This method has proven sensitive to 10 minutes of acid etching on tooth enamel, which is a lesion so incipient that is not likely to be detected in clinical practice even by a trained dentist, besides it is also sensitive to the erosion progression.

In conclusion, even though it has never been tested in a clinical trial, the highlights of the method such as: non-contact non-destructive cost-effective in theory makes it ideal for the clinical practice.
